# Finely tuned ionizable lipid nanoparticles for CRISPR/Cas9 ribonucleoprotein delivery and gene editing

**DOI:** 10.1186/s12951-024-02427-2

**Published:** 2024-04-12

**Authors:** San Hae Im, Mincheol Jang, Ji-Ho Park, Hyun Jung Chung

**Affiliations:** 1 Department of Biological Sciences, Korea Advanced Institute of Science and Technology (KAIST), 291, Daehak-ro, Yuseong-gu, Daejeon, 34141 Republic of Korea; 2 Department of Bio and Brain Engineering, Korea Advanced Institute of Science and Technology (KAIST), 291, Daehak-ro, Yuseong-gu, Daejeon, 34141 Republic of Korea; 3 KAIST Institute for Health Science and Technology, Korea Advanced Institute of Science and Technology (KAIST), 291, Daehak-ro, Yuseong-gu, Daejeon, 34141 Republic of Korea

**Keywords:** CRISPR/Cas9, Lipid nanoparticles, Ribonucleoprotein, In vivo delivery

## Abstract

Nonviral delivery of the CRISPR/Cas9 system provides great benefits for in vivo gene therapy due to the low risk of side effects. However, in vivo gene editing by delivering the Cas9 ribonucleoprotein (RNP) is challenging due to the poor delivery into target tissues and cells. Here, we introduce an effective delivery method for the CRISPR/Cas9 RNPs by finely tuning the formulation of ionizable lipid nanoparticles. The LNPs delivering CRISPR/Cas9 RNPs (CrLNPs) are demonstrated to induce gene editing with high efficiencies in various cancer cell lines in vitro. Furthermore, we show that CrLNPs can be delivered into tumor tissues with high efficiency, as well as induce significant gene editing in vivo. The current study presents an effective platform for nonviral delivery of the CRISPR/Cas9 system that can be applied as an in vivo gene editing therapeutic for treating various diseases such as cancer and genetic disorders.

**Supplementary Information:**

The online version contains supplementary material available at 10.1186/s12951-024-02427-2.

## Introduction

The development of the clustered regularly interspaced short palindromic repeat (CRISPR)-associated protein (CRISPR/Cas) system has been introduced as a powerful tool for genome engineering [1–3]. Particularly, the CRISPR/Cas9 systems has been shown promising as a cure for genetic diseases [4–6] and cancer [7, 8], with several clinical trials ongoing. Until now, the majority of the trials involve delivery of the ribonucleoprotein (RNP) including the Cas9 endonuclease and single guide-RNA (sgRNA), however its poor delivery in vivo has caused hurdles in its development as a biopharmaceutical [2]. Various delivery platforms, such as inorganic nanoparticles [9–14], polymers [15–19], and lipids [20–23] have been developed and demonstrated as effective non-viral carriers of the CRISPR-Cas9 system. However, inorganic nanoparticles confront challenges in the incomplete excretion and inducing kidney toxicity, while polymer-based systems show limited efficiency in gene editing for RNPs [19]. On the other hand, lipid nanoparticles (LNPs) show great advantages for in vivo delivery due to the facile preparation, efficient delivery, and biocompatibility [23].

LNPs have been widely investigated for the delivery of nucleic acids including siRNA and mRNA [24–27], resulting in great success in clinical trials, such as the recent cases of the approved COVID-19 vaccines BNT162b and mRNA-1273 [28, 29]. Besides nucleic acid delivery, LNPs have also shown potential for the delivery of biomolecule complexes with negative net charges, such as CRISPR/Cas RNPs [30, 31], proteins fused with negatively charged peptides [32, 33] and oligonucleotide-conjugated protein [34] Generally, the electrostatic interaction between the anionic nucleic acids and cationic lipids is crucial in forming LNPs [35]. However, for the case of RNPs, especially of the CRISPR/Cas9 system, loading the cargo into the LNPs is more complex due to the large size and cationic nature of the Cas9 endonuclease [30–32]. Although various attempts have been made to deliver RNPs using LNPs, the challenge in loading both the Cas9 protein and sgRNA, their co-localization in the nucleus, and preserving endonuclease function have led to only modest effects when applied as an in vivo therapeutic [21–23].

**Fig. 1:**
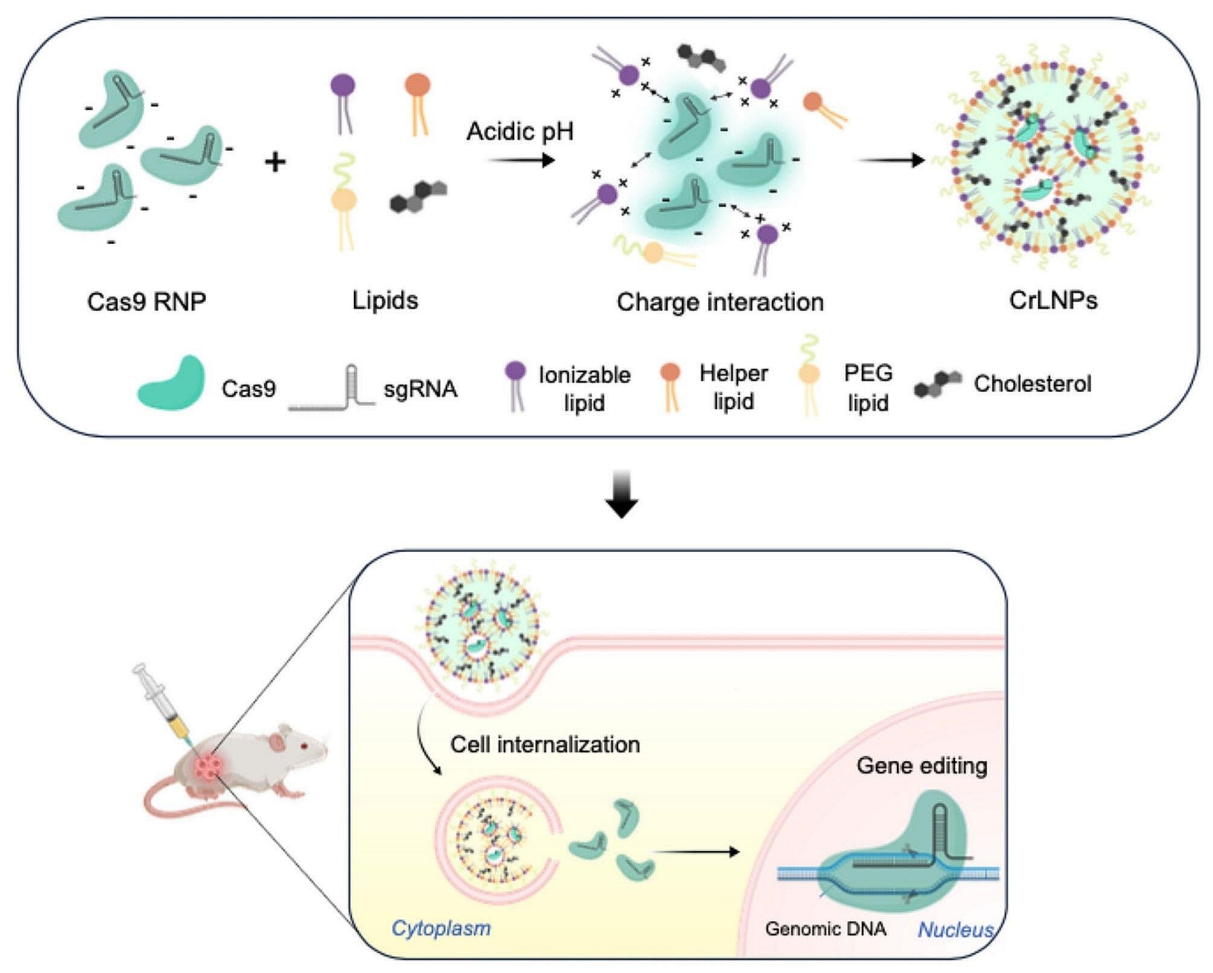
Schematic on the preparation of CrLNP for CRISPR/Cas9 RNP delivery. CrLNPs were prepared using ionizable lipids and precise control of the pH conditions, to enable efficient delivery and gene editing in target cells

Here, we developed a robust and facile method based on LNPs to effectively deliver CRISPR/Cas9 RNPs to targets cells and induce significant gene editing in vivo. An ionizable lipid-based LNP formulation was prepared for loading the Cas9 RNPs (Fig. 1). With precise control of the formulation conditions, LNPs loaded with Cas9 RNPs (CrLNPs = CRISPR/Cas9 LNPs) were successfully formed, with optimal physicochemical properties for efficient delivery while showing minimal loss in Cas9 function. The CrLNPs were able to be effectively delivered to induce gene editing in target cells, that was demonstrated for a surrogate reporter gene as well as an endogenous target gene. Furthermore, the CrLNPs were delivered in vivo and demonstrated to induce efficient gene editing in a tumor xenograft model in mice. We anticipate that our current development can provide a therapeutic platform for the treatment of intractable diseases such as cancer and genetic disorders.

## Results and discussion

### Preparation and characterization of CrLNPs

**Fig. 2:**
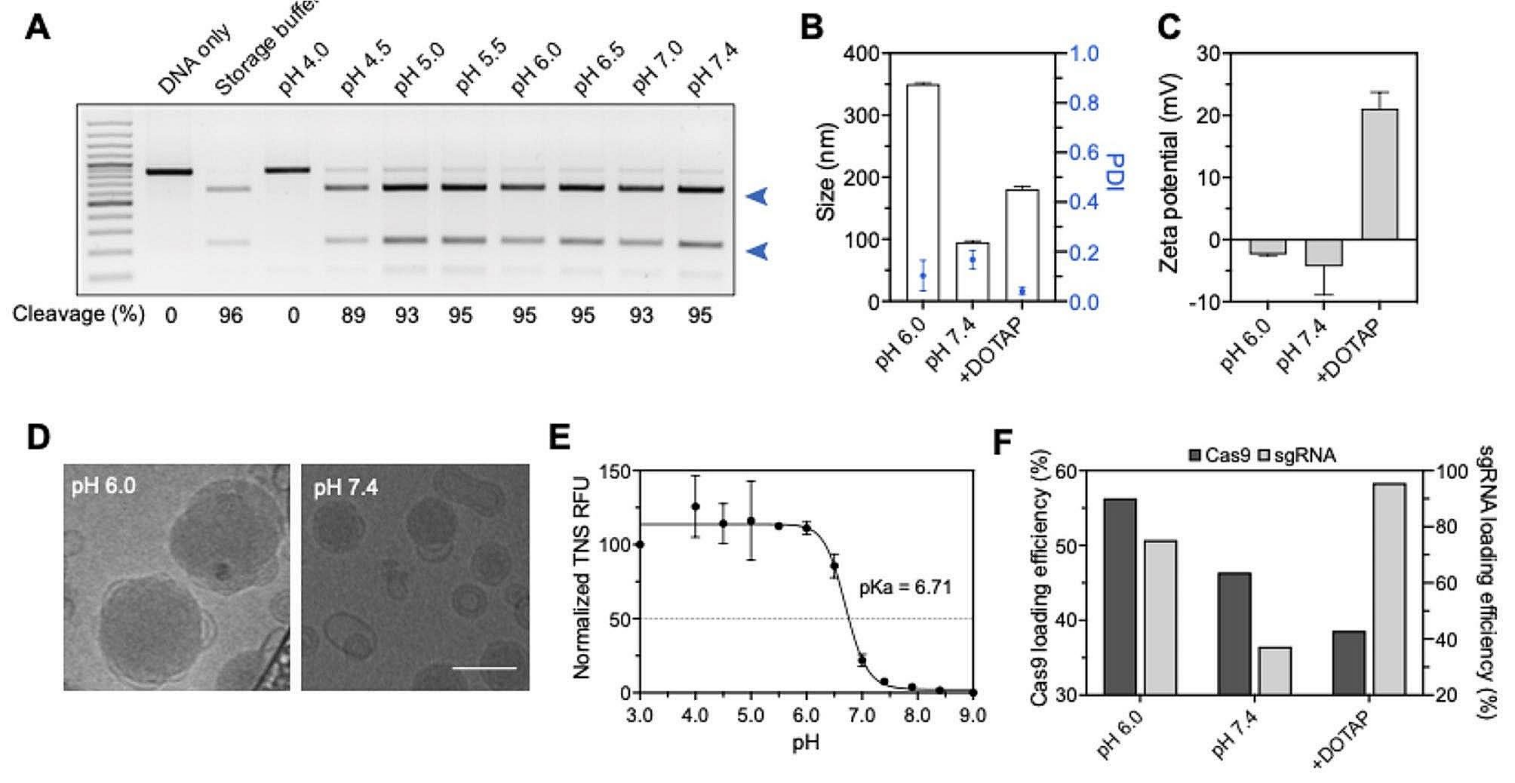
Characterization of CrLNPs. **A** Cleavage activities of Cas9 stored at various pHs, by adding the RNP with target DNA (target: *EGFR*). **B** Hydrodynamic size, **C** zeta potential, and **D** cryo-TEM of CrLNPs prepared at different pHs (pH 6.0 and 7.4). Scale bar, 100 nm. **E** TNS assay of CrLNP prepared at pH 6.0. **F** Loading efficiency of Cas9 and sgRNA in CrLNPs prepared at different pHs (pH 6.0 and 7.4)

We prepared CrLNPs using precise formulation conditions for maximizing delivery efficiency while preventing protein denaturation to preserve endonuclease function. In general, ionizable lipid-based LNPs for nucleic acid delivery are prepared under highly acidic conditions (∼ pH 4.0), that are lower than the pKa value of ionizable lipid. However, in the case of RNPs, the low-pH conditions can cause denaturation of the labile protein, resulting in loss of activity. Here, we hypothesized that the precise control of the pH condition during LNP preparation can enable the loading of Cas9 RNPs into the LNPs with high efficiency, by providing an environment in which the net charge of the RNPs is sufficient to interact with the ionizable lipid, while and preserving endonuclease function. Cas9 from *Streptococcus pyogenes* was expressed in *E.coli* and purified by FPLC (Additional file 1: Figure S1B). sgRNAs were designed to target a surrogate reporter gene, that expresses EGFP by an in-frame shift mutation upon editing of the *L858R* mutation in the *EGFR* gene(Additional file 1: Figure S2A, B) [36]. We then measured the DNA cleavage activity of Cas9 after storage at various pH conditions. Figure 2 A shows that the cleavage activity is greatly reduced when stored at more acidic pH conditions and completely lost at pH 4.0. Since pH conditions of 5.5 or above did not significantly affect Cas9 function (≥ 95% cleavage), we set these conditions as the range for CrLNP preparation.

The ionizable lipid DLin-MC3-DMA (MC3) was used for preparation of CrLNPs. The standard method for preparation of MC3-based LNPs involves an acidic pH of ∼ 4.0 [37]. Since such pH conditions would cause denaturation of the Cas9 protein, we attempted the use of a slightly acidic condition, pH 6.0, for LNP preparation. LNPs including the cationic lipid DOTAP have been reported to enable efficient delivery of RNPs in the previous study [31], and therefore used to compare with the LNPs formed by pH control (Additional file 1: Figure S3). The hydrodynamic size of the CrLNPs measured by dynamic light scattering (DLS) was ∼ 95.2 nm when prepared at pH 7.4, and increased to ∼ 350.2 nm for ones prepared at pH 6.0 (Fig. 2B). The zeta potential of CrLNPs prepared at pH 7.4 and 6.0 were − 4.3 mV and − 2.4 mV, respectively, showing close to neutral charges (Fig. 2C). LNPs including DOTAP had a size of 180.6 nm, and zeta potential of + 21.1 mV, that is due to the strong interaction of the permanent cationic lipid and RNPs. The larger size of CrLNPs prepared at pH 6.0 compared to CrLNPs prepared at pH 7.4 and added with DOTAP is probably due to the higher loading efficiency of RNPs at pH 6.0, caused by the stronger charge interaction of the cationic ionizable lipids at pH 6.0 with the anionic RNPs (Additional file 1: Figure S4). Cryo-TEM of the CrLNPs prepared at pH 6.0 revealed a particle size of ∼ 200 nm, that was comparable to the DLS measurements (Fig. 2D). On the other hand, CrLNPs prepared at pH 7.4 had irregular shapes that were polydisperse (Additional file 1: Figure S5), demonstrating the high structural stability of CrLNPs prepared at pH 6.0. Analysis of the pKa value by the TNS assay showed a value of 6.71 (Fig. 2E), that was consistent with the previous reports on MC3-based LNPs [38]. These results demonstrate that by simply changing the pH conditions, the characteristics of RNP-encapsulating LNPs are controlled and uniform CrLNPs are successfully synthesized at pH 6.0.

Loading efficiencies of Cas9 and sgRNA in CrLNPs prepared at pH 6.0 were 56.3% and 75.3% for Cas9 and sgRNA, respectively, that were higher than those in CrLNPs prepared at pH 7.4 (46.4% for Cas9, 37.3% for sgRNA) (Fig. 2F and Additional file 1: Figure S6). On the other hand, LNPs added with DOTAP showed dramatically higher loading of sgRNA (95.6%), while the loading of Cas9 was lower compared to LNPs without DOTAP. These results demonstrate that high loading of both Cas9 and sgRNA can be achieved by adjusting the pH conditions, suggesting that a slightly acidic pH can induce a sufficiently strong charge interactions between the ionizable lipids and anionic RNPs. We assumed that higher loading of the RNPs would result in higher efficiency of gene editing in cells.

### Effect of pH control on CrLNPs cellular uptake and gene editing

**Fig. 3:**
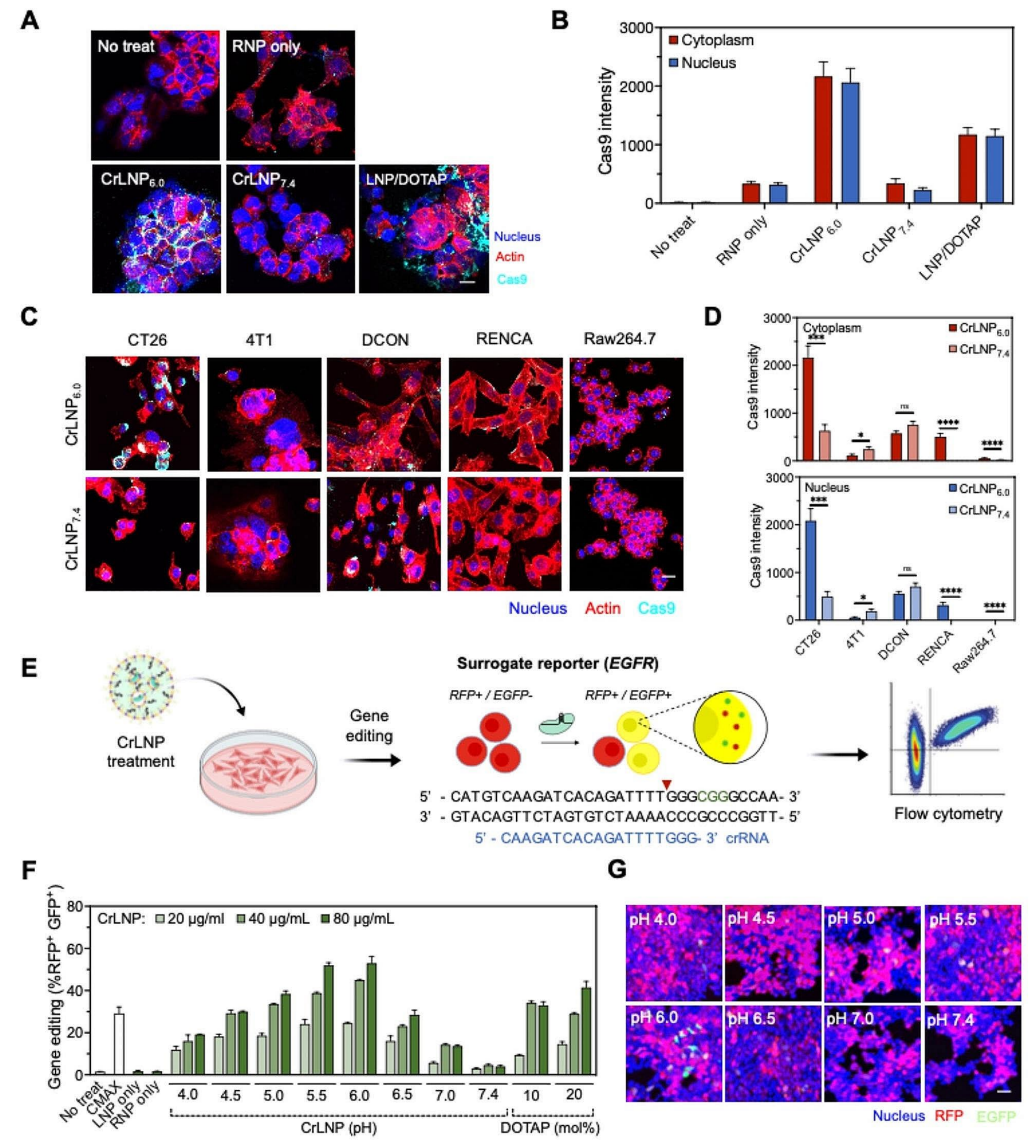
Effect of pH on the uptake and gene editing efficiency of CrLNPs. Confocal microscopy of **A**, **B** HEK293T cells and **C**, **D** various cell lines (CT26, 4T1, DCON, RENCA, Raw264.7) treated with CrLNPs to examine cellular uptake (scale bar: 10 μm for **A**, 40 μm for **C**). **B**, **D** Quantification of fluorescence by image analyses. Data are shown as mean ± SD, *P < 0.05, ***P < 0.001, ****P < 0.0001 by student’s *t*-test. **E** Schematic of CrLNPs treatment to HEK293T cells expressing surrogate reporter and analyses by flow cytometry. Gene editing efficiencies of HEK293T determined by **F** flow cytometry and **G** confocal microscopy. Scale bar, 40 μm

We examined the effect of pH condition during CrLNPs preparation on the delivery and gene editing in cells. We first treated the CrLNPs to HEK293T cells, and determined the extent of cellular uptake by confocal microscopy. CrLNPs prepared at pH 6.0 (CrLNP_6.0_) showed more efficient uptake of the Cas9 protein into both the cytoplasm and nucleus compared to ones prepared at pH 7.4 (CrLNP_7.4_) (Fig. 3A). Quantification of fluorescence intensities revealed ∼ 6.3-fold and ∼ 9.0-fold higher uptake of Cas9 delivered by CrLNP_6.0_ into the cytoplasm and nucleus, respectively, compared to CrLNP_7.4_ (Fig. 3B). The average uptake of Cas9 by CrLNP_6.0_ was ∼ 1.8-fold higher in the cytoplasm and nucleus compared to LNPs including DOTAP. Intense signals were shown for LNP/DOTAP in the extracellular regions, that can be due to the strong cationic property of the lipid, causing them to bind to the cell membrane even before internalization. Western blot analyses also showed similar results compared to confocal microscopy (Additional file 1: Figure S7). We then evaluated the delivery potential of CrLNPs in various cell lines. CrLNP_6.0_ was able to deliver Cas9 with significantly higher efficiency than CrLNP_7.4_ in CT26, RENCA, and Raw264.7 cells (Fig. 3C, D and Additional file 1: Figure S8). Examining cytotoxicity demonstrated that CrLNP_6.0_ treatment did not result in significant cytotoxicity across various cell lines, including CT26, RENCA, and Raw264.7 cells (Additional file 1: Figure S9). On the other hand, CrLNP_6.0_ did not show any significant enhancement in Cas9 uptake in the cytoplasm and nucleus of 4T1 and DCON cells. These results demonstrate that pH-controlled Cas9-LNPs can efficiently deliver Cas9 into the cytoplasm and nucleus of several cell types, but could not be observed in certain cell lines.

Next, we assessed the gene editing efficiency of CrLNPs in a surrogate reporter system. HEK293T cells expressing the surrogate reporter were treated with CrLNPs and analyzed by flow cytometry and confocal microscopy (Fig. 3E). As the pH increased from 4.0 to 6.0 during CrLNP preparation, the gene editing efficiency gradually increased from 19.2 to 53.0% with 80 µg/mL CrLNP treatment (Fig. 3F). However, the efficiency suddenly decreased for CrLNPs prepared at pH 6.5 or above. Confocal microscopy of treated cells also revealed a pattern similar to the flow cytometry results (Fig. 3G). These results confirm that strongly acidic conditions (∼ pH 4.0) during CrLNP preparation cause severe loss of Cas9 endonuclease function, with activity gradually recovering with increasing pH. However, when the pH is close to neutral, that is higher than the pKa value of the ionizable lipid (6.44), the gene editing efficiency is rather reduced due to the small loading amount of Cas9 and sgRNA, as described by the results in Fig. 2F.

### Validation and optimization of CrLNPs

**Fig. 4:**
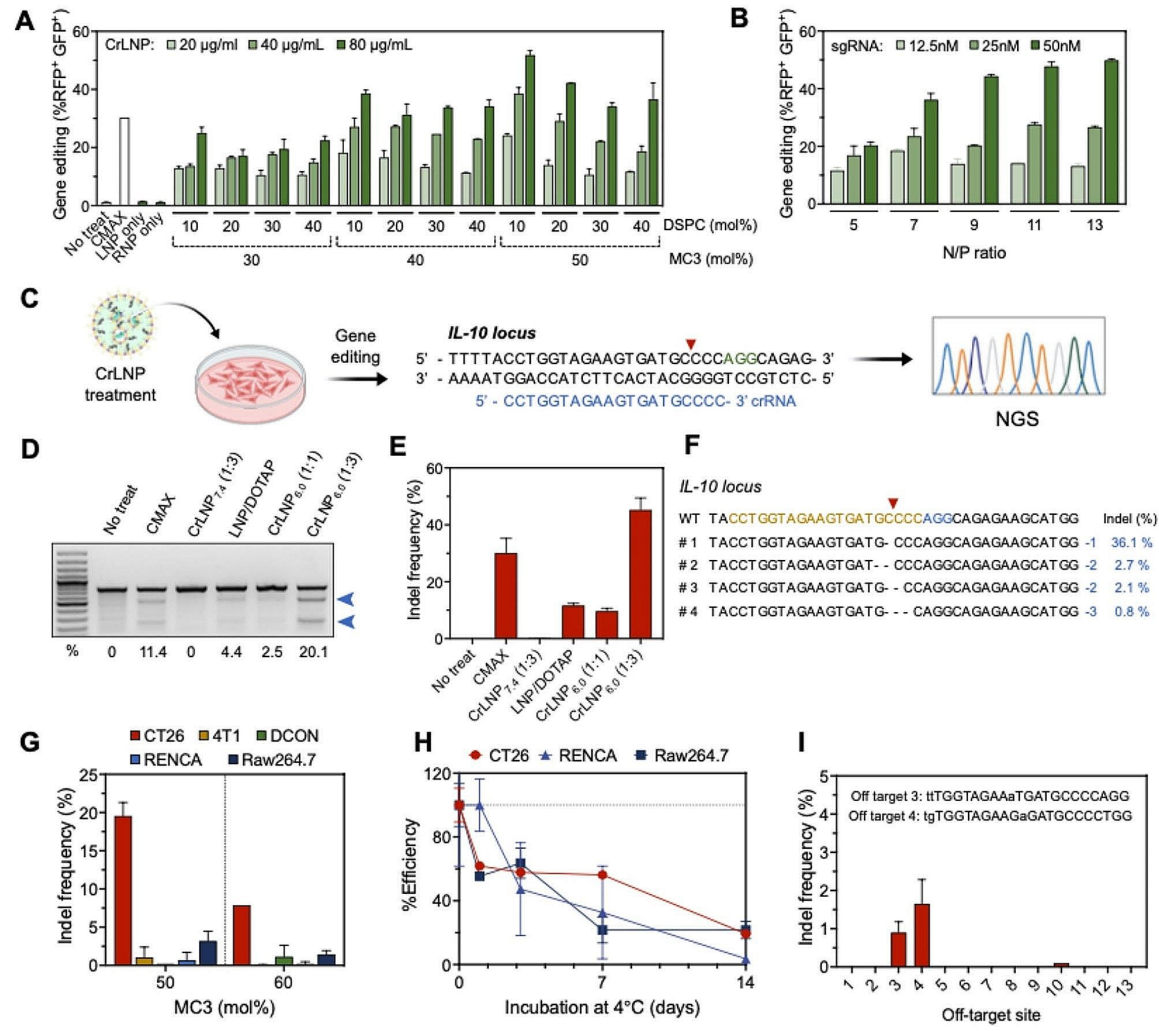
Validation and optimization CrLNP_6.0_ on gene editing in vitro. Gene editing efficiencies by treating cells with CrLNPs targeting the surrogate reporter gene under various **A** lipid compositions, and **B** N/P ratios. **C** Scheme of endogenous gene (*IL-10*) editing in various cell lines analyzed by targeted deep sequencing. Indel frequencies of CT26 cells by treating with CrLNPs prepared at different Cas9 to sgRNA molar ratios (1:1, or 1:3) analyzed by **D** T7E1 assay and **E** NGS. **F** Representative sequence data from **E** for CrLNP_6.0_ (orange: crRNA, blue: PAM, -: deletion, WT: wild-type). **G** Indel frequencies of various cell lines treated with CrLNP_6.0_ at different molar ratios of MC3. **H** Changes in efficiencies of gene editing in cells treated with CrLNP_6.0_ stored at 4℃ for various incubation times. **I** Indels at possible off-target sites in CT26 cells treated with CrLNP_6.0_

We next examined the effect of varying lipid composition of the CrLNPs on gene editing. First, we varied the molar ratio of the ionizable lipid MC3 and helper lipid 1,2-distearoyl-sn-glycero-3-phosphocholine (DSPC), that are known for their major roles in cellular transfection [39]. Treating the CrLNPs at various ratios of MC3 to HEK293T surrogate reporter cells resulted in an increase in gene editing efficiency as the MC3 ratio was increased and the DSPC ratio decreased (Fig. 4A). This can be due to the stronger packaging, charge, and endosomal escape of the LNPs with a higher content of MC3. When the DSPC ratio was increased, the gene editing efficiencies decreased, especially at 50% MC3 (51.9% at 10% DSPC vs. 36.7% at 40% DSPC). This can possibly be because the molar ratio of cholesterol, that is related to LNP stability, was decreased as the ratio of DSPC was increased. We also attempted varying the N/P ratio of the CrLNPs calculated based on the number of phosphate groups within the sgRNA. Increasing the N/P ratios of the CrLNPs from 5 to 13 resulted in a gradual increase in gene editing efficiency at high concentrations of sgRNA, and plateaued at an N/P ratio of 11 and above (Fig. 4B). We next evaluated gene editing of an endogenous gene, *IL-10*, an anti-inflammatory cytokine used for various therapeutic purposes (Fig. 4C). sgRNAs designed for each target gene were synthesized by in vitro transcription (Additional file 1: Figure S10A-C). Measuring the DNA cleavage activity of the twelve sgRNAs designed for the *IL-10* gene showed that sgRNA-5 resulted in the highest efficiency in cleavage (Additional file 1: Figure S10D). Furthermore, gene editing efficiencies by the sgRNAs targeting *IL-10* were determined by treating to various cell lines, with sgRNA-5 showing significant gene editing efficiencies in most of the cell lines **(**> 7.3% indels in 5 out of 6 cell lines; Additional file 1: Figure S11). Treating the CrLNPs prepared at various Cas9 to sgRNA molar ratios to CT26 cells revealed that CrLNP_6.0_ induced gene editing with a 2-fold increase in efficiency compared to CMAX, according to the T7 Endonuclease I (T7E1) assay results (Fig. 4D). Since the T7E1 assay is limited in accurate analysis of indel value [40], we performed targeted deep sequencing of the treated cells, showing an indel frequency of 45.2% by CrLNP_6.0_ (Fig. 4E). The representative sequences after gene editing of *IL-10* by CrLNP_6.0_ are shown in Fig. 4F. The effect on hemolysis was reduced for CrLNP_6.0_ compared to CrLNP_7.4_ (Additional file 1: Figure S12), meaning that endosome escape did not directly affect gene editing efficiency. Instead, the higher loading of Cas9 and sgRNA in the CrLNP_6.0_ compared to CrLNP_7.4_ could be the main reason for the enhancement in gene editing. Based on these results, an MC3 ratio of 50%, DSPC ratio of 10%, and an N/P ratio of 11 was found as the optimal composition of the CrLNPs leading to most effective delivery and gene editing, and therefore this condition was selected for further experiments.

We then attempted to apply the optimized CrLNPs to other ionizable lipids with high transfection potential. When equivalent CrLNPs formulations were prepared using ALC-0315 or SM-102 instead of MC3, the hydrodynamic sizes of both formulations appeared smaller than the original MC3-LNPs, and was less effective in gene editing (< 5% indels) (Additional file 1: Figure S13A, B). We also validated gene editing by CrLNP_6.0_ in various cell lines, demonstrating that the most efficient gene editing occurred in CT26 cells, ∼ 19.6%, at an MC3 ratio of 50% (Fig. 4G). Raw264.7 cells showed the second highest indel frequencies of ∼ 3.2%. We determined the stability of CrLNP_6.0_ by incubation at 4℃ and measuring the indel frequencies according to various incubation times. Figure 4H shows that the indel frequency decreased 61.9% in CT26 cells after storage for 1 day, and decreased ∼ 56.3% and ∼ 19.3% after 7 days and 14 days at 4℃, respectively. These results indicated that CrLNP_6.0_ should be used within 1 day to get efficient gene editing. Off-target effects by the CrLNPs were also examined by selecting 13 potential off-target sites that can be targeted by *IL-10* sgRNA and analysis by targeted deep sequencing. Results showed that the treatment of CrLNP_6.0_ targeting *IL-10* to CT26 cells can induce indels at only 3 sites among the 13 potential off-target sites, at low efficiencies (> 1.5%) (Fig. 4I). These results confirm that the pH-controlled, MC3-based LNPs are an effective formulation for RNP delivery and gene editing in target cells, with a low chance of off-target effects.

### In vivo **delivery of CrLNPs**s

**Fig. 5:**
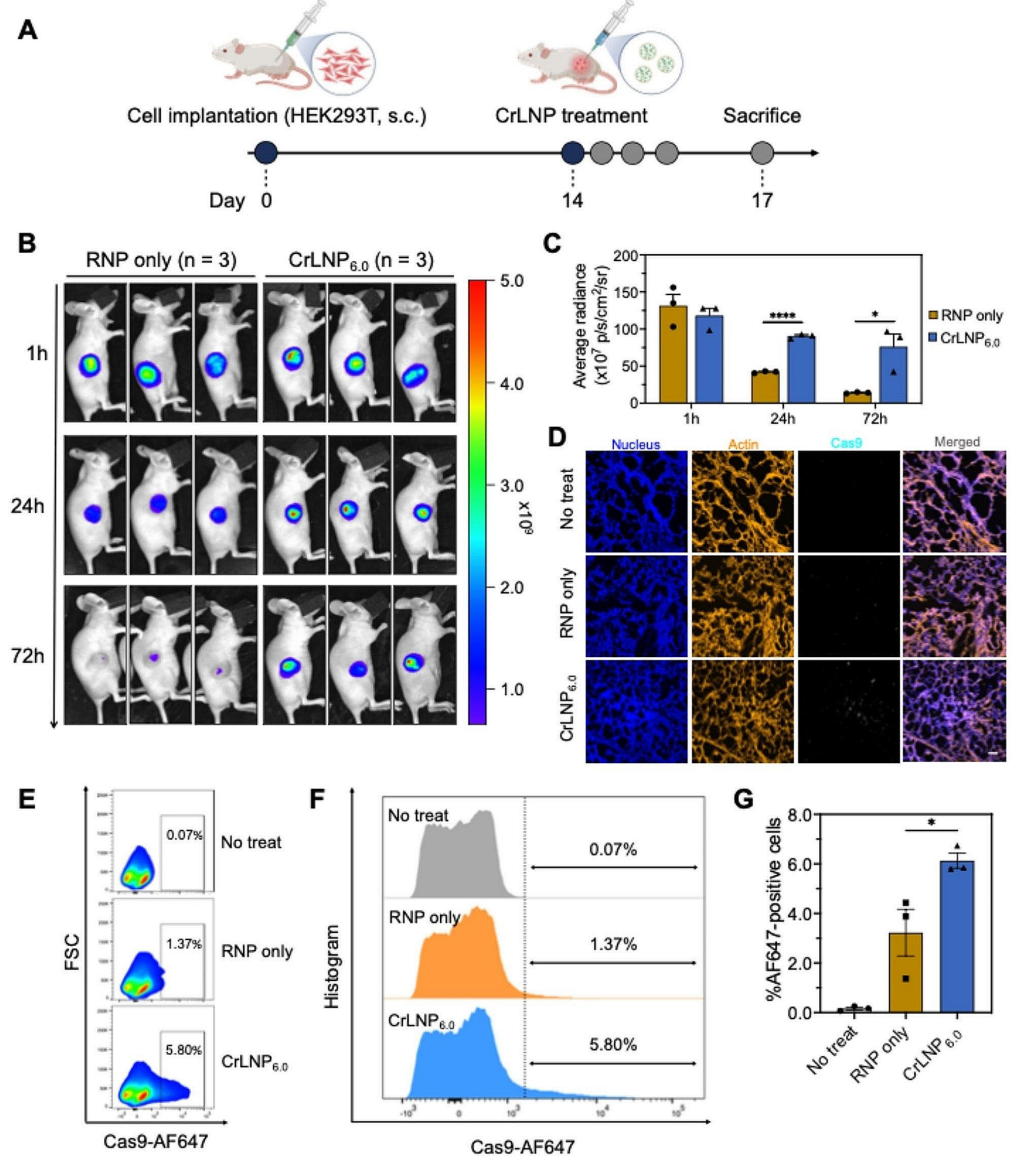
In vivo delivery of CrLNPs. **A** Scheme on the treatment of CrLNP_6.0_ to HEK293T xenograft mouse model. CrLNP_6.0_, including AF647-conjugated Cas9, was treated for fluorescence imaging. **B** IVIS imaging at different time points after intratumoral injection of CrLNP_6.0_, and **C** quantification of signals (*n* = 3). Data are shown as mean ± SD, *P < 0.05, ****P < 0.0001 by student’s *t*-test. **D** Confocal microscopy of tumor tissues 72 h after injection of CrLNP_6.0_. Scale bar, 40 μm. Flow cytometry of cells from tumor tissues 72 h after injection with CrLNP_6.0_, shown by **E** scatter plot and **F** histogram of representative samples for each group, as well as **G** mean values for each group (*n* = 3). Data are shown as mean ± SD, *P < 0.05 by one-way ANOVA.

We evaluated the potential of CrLNP_6.0_ for the delivery of Cas9 RNPs in vivo in a tumor xenograft model in mice. HEK293T cells expressing the surrogate reporter were subcutaneously implanted into mice, and CrLNP_6.0_ including AF647-conjugated Cas9 was locally administered by intratumoral injection. and AF647-conjugated Cas9 protein fluorescence intensity was obtained using in vivo optical system (IVIS) (Fig. 5A). The treatment of naked RNP resulted in a rapid decay in Cas9 fluorescence over time, with a ∼ 68.0% decrease in signal after 24 h, and ∼ 89.3% decrease after 72 h (Fig. 5B, C). On the other hand, delivery by CrLNP_6.0_ resulted in sustained signals of Cas9 that were comparable to CrLNP_4.0_ (Additional file 1: Figure S14), with ∼ 76.6% of the signal remaining after 24 h, and ∼ 64.4% after 72 h. No significant leakage to other organs was observed (Additional file 1: Figure S15). This can be because CrLNP_6.0_ not only enables efficient delivery into cells, but also allows penetration into the HEK293T tumors. Three days after the injection, mice were sacrificed and tumor tissues were harvested for further analyses. Microscopic observation of the tumor tissues indeed showed the presence of Cas9 signals for CrLNP_6.0_, proving the efficient tissue penetration and cellular uptake in the tumor (Fig. 5D). Quantification of the signals by flow cytometry analysis also confirmed the imaging and microscopy results, with an average of ∼ 6.1% of AF647-positive cells for CrLNP_6.0_, that was significantly higher than naked RNPs (∼ 3.2%) (Fig. 5E-G). These results demonstrate the potency of CrLNP_6.0_ as a delivery formulation of Cas9 RNPs into tumor tissues.

**Fig. 6:**
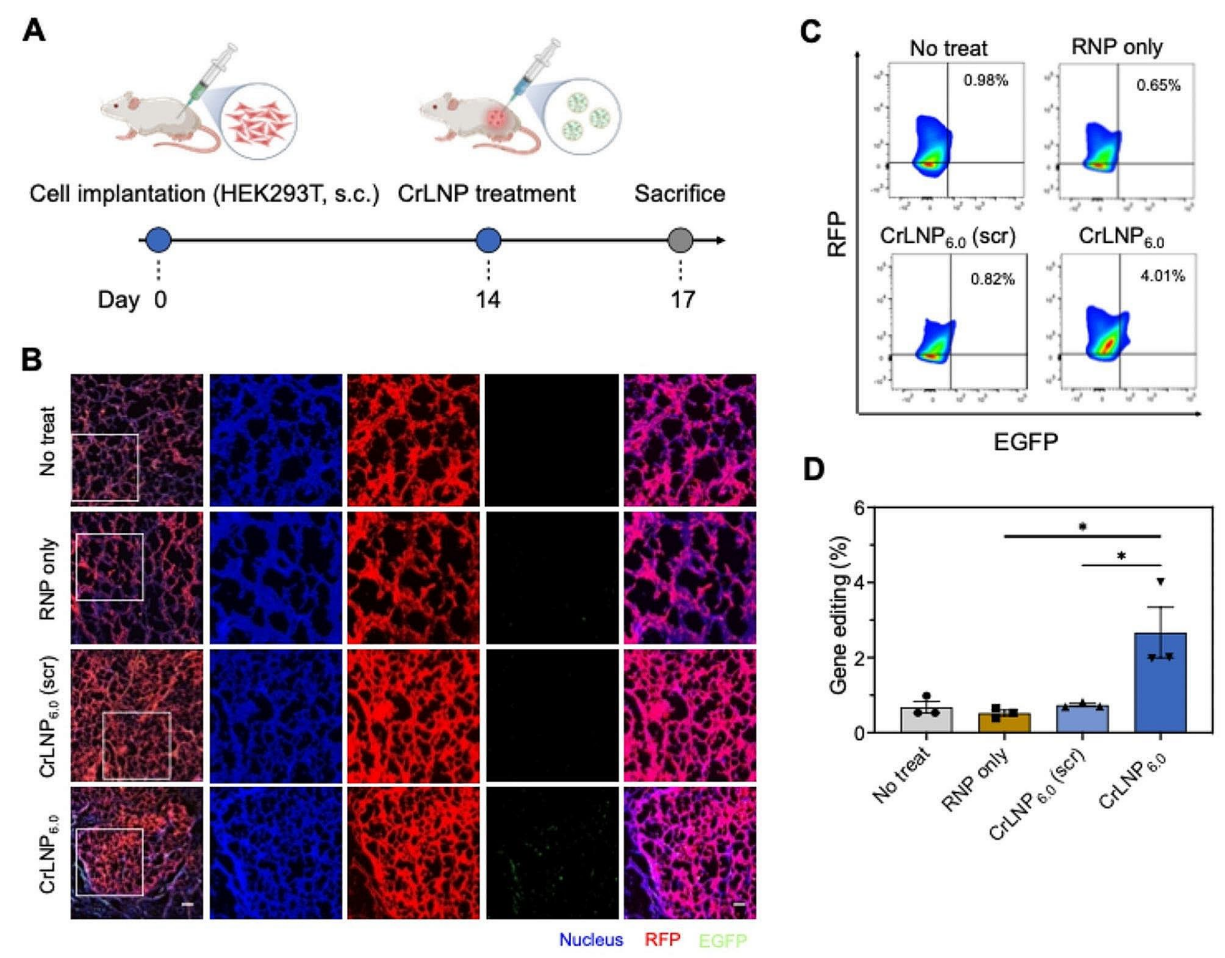
In vivo gene editing by treatment of CrLNP_6.0_. **A** Scheme on the treatment of CrLNP_6.0_ targeting the surrogate reporter to the HEK293T xenograft mouse model. **B** Confocal microscopy of tumor tissues 72 h after intratumoral injection of CrLNP_6.0_. Scale bar, 40 μm. Gene editing efficiencies measured by flow cytometry of cells from tumor tissues 72 h after injection, shown as **C** scatter plot of a representative sample from each group, and **D** mean values for each group (*n* = 3). Data are shown as mean ± SD, *P < 0.05 by one-way ANOVA

We evaluated the CrLNPs in inducing gene editing efficiency in HEK293T tumors in vivo (Fig. 6A). Intratumoral injection of CrLNP_6.0_ and the control formulations did not cause significant differences in the sizes of tumors between the treatment groups, indicating that no serious side effects occurred (Additional file 1: Figure S16). Observing the EGFP fluorescence of the harvested tumor tissue three days after injection by confocal microscopy as well as ex vivo tissue imaging obviously showed the presence of EGFP signals in the tumor tissue treated by CrLNP_6.0_, while the other treatment groups did not show any signals (Fig. 6B and Additional file 1: Figure S17). According to flow cytometry analysis, ∼ 2.7% of GFP-positive cells were detected in the tumors for the CrLNP_6.0_ treatment group, by gene editing of the surrogate reporter (Fig. 6C, D). On the other hand, naked RNPs and CrLNP_6.0_ (scr) did not induce significant gene editing (< 0.74%), and CrLNP_4.0_ exhibited lower gene editing efficiency compared to CrLNP_6.0_ (Additional file 1: Figure S18), confirming the specificity of targeted gene editing in vivo. Apparently, we find that in vivo delivery of Cas9 RNPs by CrLNP_6.0_ enables efficient tissue penetration and cellular uptake, leading to significant gene editing in tumors. Therefore, we anticipate that the current development can provide a powerful tool for therapeutic applications involving hard-to-transfect tissues.

## Conclusion

Here, we introduced CrLNPs as an effective nano-delivery platform for the CRISPR/Cas9 system. We were able to efficiently load Cas9 RNPs into ionizable LNPs by precisely controlling the pH conditions during LNP preparation. CrLNPs could effectively deliver the RNPs to various target cells, leading to substantial gene editing in vitro and in vivo. Our strategy can be versatilely applied to various therapeutic proteins that are hard-to-deliver or show low efficacy in vivo. Expanding the strategy to various LNP formulations and further optimization for the delivery into various cells, including primary cells, should be performed in the future. We anticipate that the current development provides a promising platform that can be applied to treat various diseases, such as cancer and genetic disorders.

## Methods

### Materials

(6Z,9Z,28Z,31Z)-Heptatriaconta-6,9,28,31-tetraen-19-yl 4-(dimethylamino)butanoate (DLin-MC3-DMA), 6-((2-hexyldecanoyl)oxy)-N-(6-((2-hexyldecanoyl)oxy)hexyl)-N-(4-hydroxybutyl)hexan-1-aminium (ALC-0315), methoxypolyethyleneglycoloxy(2000)-N,N-ditetradecylacetamide (ALC-0159), and heptadecan-9-yl 8-((2-hydroxyethyl)(6-oxo-6-(undecyloxy)hexyl)amino)octanoate (SM-102) were purchased from MedKoo Biosciences. Cholesterol, 1,2-distearoyl-sn-glycero-3-phosphocholine (DSPC), 1,2-dimyristoyl-rac-glycero-3-methoxypolyethylene glycol (DMG-PEG, MW 2000), and 1,2-dioleoyl-3-trimethylammonium-propane (DOTAP) were obtained from Avanti Polar Lipids. Sodium citrate tribasic dihydrate and citric acid were purchased from Sigma. Phosphate buffered saline (PBS) was purchased from Cytiva.

### Cas9 purification

Cas9 expression plasmid pET28a-Cas9-His **(**Addgene, Additional file 1: Figure S1A) was transformed to *E.coli* Rosetta (DE)3 cells using the heat shock method. Transformed bacterial colonies were selected in LB agar containing ampicillin (100 µg/mL), and confirmed by colony PCR. Transformed cells were incubated in LB media containing ampicillin (100 µg/mL) until OD600 reached 0.8, and added with 0.5 mM of isopropyl β-D-1-thiogalactopyranoside (IPTG) for induction at 18℃ for 16 h with gentle agitation. Then, cells were collected and lysed in lysis buffer (50 mM Na_2_PO_4_, 300 mM NaCl, 10 mM imidazole, 1% TritonX-100, 0.5 mM PMSF, 1 mM DTT, 1 mg/mL lysozyme, 16.7 U/mL benzonase, pH 8.0) with sonication. Lysates were cleared by centrifugation (13,000 g, 30 min) and passed through a 0.2 μm syringe filter. His-trap^TM^ HP (GE Healthcare) and HiLoad Superdex 200 26/600 (GE Healthcare) chromatography were performed using ÄKTAprime Plus (GE Healthcare). Protein were eluted using elution buffer (50 mM Na_2_PO_4_, 300 mM NaCl, 250 mM imidazole, 0.05% β-mercaptoethanol, pH 8.0), and dialyzed against storage buffer (50 mM Tris-Cl, 200 mM KCl, 0.1 mM EDTA, 1 mM dithiothreitol (DTT), 0.5 mM PMSF, 20% glycerol, pH 8.0) using Slide-A-Lyzer (MWCO: 20 kDa, Thermo Scientific). For Alexa Fluor 647 NHS ester (succinimidyl ester) (AF647, Thermo Fisher Scientific) conjugation, the purified Cas9 was concentrated using Amicon centrifugal filters (MWCO 30 kDa, Merck/MilliporeSigma), dialyzed with PBS (pH 8.0) and reacted with AF647 (molar ratio of Cas9:AF647 = 1:1) for 2 h at room temperature with shaking at 500 rpm. After the reaction, the labeled Cas9 was dialyzed with storage buffer and stored at -80 ℃.

### sgRNA synthesis

Double-strand DNA (dsDNA) templates were synthesized with forward template DNA including T7 promoter and 20 bp crRNA sequence, reverse template DNA, Power-Pfu (500 U/µL, NanoHelix), and dNTPs (10 mM, Thermo Scientific). sgRNAs were synthesized by in vitro transcription using the dsDNA template, T7 RNA polymerase (50,000 units/mL, NEB), 50 mM MgCl_2_, 0.1 M DTT, rNTPs (100 mM, Jena Bioscience), and RNase inhibitor murine (40,000 units/mL, NEB) at 37 ℃ for 16 h, precipitated with isopropanol (Sigma) and purified with GeneAll ExpinTM PCR SV kit (GeneAll Biotechnology). sgRNA concentration was measured using NanoDrop 2000 (Thermo Scientific), and characterized by 1.0% denaturing formaldehyde gel electrophoresis with MOPS buffer (Biosolution). sgRNA was freeze-dried using HyperVAC VC2124 (Hanil Scientific) for long-term storage.

### Preparation and characterization of CrLNPs

CrLNPs were synthesized by the ethanol mixing method using Ignite (Precision Nanosystems) at a flow rate of 12 mL/min. All lipids were mixed in a glass vial with specified molar ratios and dried overnight. The molar ratio of DLin-MC3-DMA/DSPC/Cholesterol/DMG-PEG was 50/X/38.5-X/1.5, where X represents the molar ratio of DSPC. Cas9 protein and sgRNA were mixed in PBS at a certain pH for 10 min at room temperature to form RNPs. Lipid films were dissolved in ethanol and rapidly mixed with RNPs dissolved in PBS (1:3, v/v). After 10 min at room temperature, the resultant CrLNPs were dialyzed with Slide-A-Lyzer (MWCO: 20 kDa) for 2 h at 4 ℃. The hydrodynamic size and zeta potential of CrLNPs were measured using ELSZ-2000ZS (Otsuka). Cryo-TEM and standard TEM were performed using Tecani G2 Spirit TWIN (FEI). Encapsulation efficiencies of Cas9 and sgRNA were analyzed using SDS-PAGE and Quant-iT RiboGreen RNA assay (Invitrogen). Hemolysis assay was performed with human RBCs (Innovative Research) suspended in PBS (pH 7.4) or citrate buffer (20 mM citrate, 130 mM NaCl, pH 5.5) and adding the CrLNPs or controls in a 96-well microplate for incubation at 37 ℃ for 1 h. The plate was centrifuged at 1,000 g for 5 min, and the supernatant was analyzed by measuring the absorbance at 540 nm using a microplate reader (Infinite M200 PRO, Tecan). The TNS assay was performed to measure the pKa value according to a previous report [41]. In brief, buffers with pHs from 3.0 to 9.0 in 0.5 increments were prepared with 20 mM citrate buffer (pH 3.0-5.5), 20 mM sodium phosphate buffer (pH 6.0–8.0), and 20 mM Tris-HCl buffer (pH 8.0–9.0) including 150 mM NaCl. 186 µL of each buffer were mixed with 2 ${\upmu }\text{L}$of 0.6 mM 6-(*p*-Toluidino)-2-naphthalenesulfonic acid sodium salt (TNS) stock solution, and added with 12 µL of CrLNPs (0.5 mM lipid) with shaking at room temperature (400 rpm) for 10 min. Using a spectrofluorometer (Molecular Devices), fluorescence was measured at λ_ex_ = 321 nm and λ_em_ = 447 nm, and pKa was determined based on the pH value at which the fluorescence intensity reached 50% of its maximum.

### DNA cleavage assay

One microgram of Cas9 stored in PBS at various pHs was mixed 120 ng of target DNA, 750 ng of sgRNA, and NEB3.1 buffer (25 mM Tris-HCl, 50 mM NaCl, 5 mM MgCl_2_, 50 µg/mL BSA, NEB) and incubated at 37 ℃ for 90 min. Then the solution was sequentially treated with RNase A (Invitrogen) and 5X stop solution (1.2% SDS, 250 mM EDTA), purified with Expin PCR SV kit (GeneAll), and characterized by agarose gel electrophoresis and imaging with ChemiDoc (Bio-Rad).

### Cell culture

The plasmid expressing the surrogate reporter gene, pMRS-CMV (Toolgen) was transfected to HEK293T cells using Lipofectamine 3000 according to the manufacturer’s instructions. RFP-positive cells were sorted using Moflo Astrios EQ (Beckman Coulter), followed by selection and culture of cells with the highest RFP signals. CT26 murine colon cancer cells, 4T1 murine breast cancer cells, RENCA murine kidney cancer cells, Raw264.7 murine macrophage cells, and HEK293T human embryonic kidney cells were obtained from the American Type Culture Collection (ATCC). DCON murine gastric cancer cells were kindly provided by Dr. Sam S. Yoon (Memorial Sloan Kettering Cancer Center) and Dr. Sandra Ryeom (University of Pennsylvania) [42]. CT26, 4T1, and RENCA cells were cultured in RPMI-1640 (Gibco) supplemented with L-glutamine, 25 mM HEPES (Gibco), 10% fetal bovine serum (FBS, Hyclone) and 1% penicillin/streptomycin (P/S, Gibco). DCON, Raw264.7, HEK293T cells were cultured in DMEM (Gibco) containing the same supplements as RPMI 1640. Cells was cultured in a CO_2_ incubator (BB15, Thermo Fisher) at 37 ℃ at 5.0% CO_2_ condition.

### Cellular uptake and cytotoxicity analyses

Cells were seeded (0.4 × 10^5^ cells/well) in an 8-well chamber slide (SPL Life Sciences) for 24 h, media was changed with fresh media, and treated with CrLNPs including AF647 labeled Cas9 for 6 h at 37 ℃. Cells were then washed 3 times with Dulbecco’s phosphate buffered saline (DPBS, Gibco) and fixed with 4% paraformaldehyde (Thermo Fisher) for 15 min at room temperature. After washing 3 times with DPBS, cells were permeabilized with 0.1% Triton-X 100 (Merck/MilliporeSigma) for 15 min at room temperature, washed with DPBS 3 times, and stained with ActinRed 555 ReadyProbes (Thermo Fisher) for 30 min. After washing with DPBS, cells were mounted with Vectashield including 4’, 6-diamidino-2-phenylindole (DAPI, Vector Laboratories) and imaged using a laser scanning confocal microscope (LSM 880, Carl Zeiss). The acquired images were processed using ZEN (Carl Zeiss) and CellProfiler (Broad Institute) software. To measure cytotoxicity, cells were seeded in 96-well microplates (1.6 × 10^4^ cells/well), treated with the CrLNPs for 6 h at 37 ℃, and added with the Cell Counting Kit-8 solution (CCK-8, Dojindo Laboratories) with incubation for 1 h at 37 ℃. Cell viability was analyzed by measuring absorbance at 450 nm.

### Flow cytometry and Western blot

Cells were treated with CrLNPs in microplates at pre-determined conditions. For flow cytometry, cells were collected by centrifugation at 300 g, for 3 min, and re-suspended in DPBS containing Hoechst 33342 (Thermo Fisher). Flow cytometry was performed using LSR Fortessa (BD Biosciences), and analyzed using FlowJo v10 (TreeStar). For western blot analysis, cells treated with CrLNPs were washed with DPBS, and lysed with RIPA buffer (Sigma) supplemented with a protease inhibitor cocktail (Cell Signaling). Lysates were centrifuged at 12,000 g for 5 min to collect the supernatant, and protein concentration was quantified by the BCA assay (Thermo Fisher). 40 µg of total protein was loaded to 7.5% SDS-PAGE and transferred to polyvinylidene difluoride (PVDF, Bio-rad) using a Trans-Blot turbo transfer system (Bio-rad). After transfer, the membrane was washed with TBST buffer (20 mM Tirs-HCl, 150 mM NaCl, 0.1% Tween-20, pH 7.5) and blocked with 5% skim milk (dissolved in TBST, Bio-rad) for 1 h at room temperature. Primary antibodies were added and left overnight at 4℃, washed 3 times with TBST, and incubated with a secondary antibody for 1 h at room temperature. After washing with TBST, ECL substrate (Bio-rad) was added, and imaged using ChemiDoc (Bio-Rad).

### Targeted deep sequencing

Cells were seeded (0.8 × 10^5^ cells/well) in 24-well microplates for 24 h. and treated with CrLNPs for 72 h at 37 ℃. The genomic DNA was isolated using DNeasy Blood & Tissue DNA kit (Qiagen), and target regions at the *IL-10* locus were amplified using specific primers (Additional file 1: Table S1), Phusion high-fidelity DNA polymerase (Thermo Fisher) and SimpliAmp Thermal Cycler (Applied Biosystems). Off-target regions for *IL-10* sgRNAs were predicted with RGEN Cas-OFFinder and amplified as mentioned above. PCR products were amplified using specific primers (Additional file 1: Table S2) and purified using Expin PCR SV kit (GeneAll), followed by targeted deep sequencing using MiniSeq (Illumina). Indel frequencies were calculated using RGEN Cas9-analyzer.

### Animal experiments

BALB/c nude mice (7 weeks old, female) were engrafted subcutaneously in the flank with surrogate reporter HEK293T cells (1 × 10^6^ cells per graft) and Matrigel (Corning) mixture. CrLNPs (30 µL) were intratumorally injected when the average tumor size reached ∼ 60 mm^3^. The day of the treatment was designated as day 0, and tumor volumes were measured using an electronic caliper and calculation based on the formula *tumor volume* = 4.19 *LWH* (*L = length of the tumor, W = width of the tumor, H = height of the tumor*). For examining in vivo delivery, CrLNPs containing AF647 conjugated Cas9 were injected intratumorally (dose: sgRNA 0.5 mg/kg), and whole body imaging was performed at pre-determined time intervals using IVIS (PerkinElmer) with the Cy5.5 channel (λ_ex_ = 580 nm, λ_em_ = 620 nm). The tumors and other organs were harvested at each time point, and imaged with IVIS or sectioned and observed by confocal microscopy. Gene editing efficiencies in tumors were analyzed by sacrificing mice at 72 h post-injection, and tumors were harvested. Tumors were minced into 1-3mm^3^ pieces for incubation with 1 mg/mL collagenase II (Sigma) and 100 Kunits/mL DNase I (Sigma) for 1 h at 37 ℃, and flowed through a 70 μm cell strainer (Corning). After incubating in ACK lysis buffer (Lonza) for 1 min, Cells were washed with DPBS and analyzed by flow cytometry.

### Statistical analysis

All data were represented as mean ± S.D. The student’s *t*-test and one-way ANOVA were used to determine statistical significance. Further analysis was performed using Prism 9 (GraphPad). *P* < 0.05 was considered statistically significant.

### Electronic supplementary material

Below is the link to the electronic supplementary material.

Supplementary Material 1

## Data Availability

All data about this study are included in this published article and its additional file.

## Abbreviations

CRISPR: Clustered regularly interspaced short palindromic repeat
CRISPR/Cas9: CRISPR-associated protein 9
dsDNA: Double-stranded DNA
sgRNA: Single guide RNA
crRNA: CRISPR RNA
RNP: Ribonucleoprotein
LNP: Lipid nanoparticle
CrLNP: CRISPR/Cas9 LNP
DSPC: 1,2-distearoyl-sn-glycero-3-phosphocholine
DOTAP: 1,2-dioleoyl-3-trimethylammonium-propane
TEM: Transmission electron microscope
Cryo-TEM: Cryogenic TEM
IL-10: Interleukin 10
CMAX: CRISPRMAX™
T7E1: T7 Endonuclease I
EGFP: Enhanced green fluorescent protein
LB: Luria-bertani broth
PCR: Polymerase chain reaction
IPTG: Isopropyl β-D-1-thiogalactopyranoside
PMSF: Phenylmethylsulfonyl fluoride
DTT: Dithiothreitol
MWCO: Molecular weight cut-off
AF647: Alexa Fluor™ 647
dNTPs: Deoxyribonucleotide triphosphates
rNTPs: Ribonucleoside triphosphates
MOPS: 3-(N-morpholino) propane sulfonic acid
BSA: Bovine serum albumin
SDS-PAGE: Sodium dodecyl-sulfate polyacrylamide gel electrophoresis
DPBS: Dulbecco’s phosphate-buffered saline
DMEM: Dulbecco’s modified eagle medium
RPMI 1640: Roswell park memorial institute 1640
DAPI: 4',6-diamidino-2-phenylindole
CCK-8: Cell counting kit-8
BCA: Bicinchoninic acid assay
TBST: Tris-buffered saline with 0.1% Tween20® detergent
ECL: Enhanced chemiluminescence
WT: Wild-type
