# Supplementary material for: Finely tuned ionizable lipid nanoparticles for CRISPR/Cas9 ribonucleoprotein delivery and gene editing
Source: J Nanobiotechnology. 2024 Apr 12;22:175. doi: 10.1186/s12951-024-02427-2 (PMC11015636; doi:10.1186/s12951-024-02427-2)
Supplement: Supplementary file 1 — Supplementary Material 1 [file 12951_2024_2427_MOESM1_ESM.docx]

**Finely tuned ionizable lipid nanoparticles for CRISPR/Cas9 ribonucleoprotein delivery and gene editing**

San Hae Im^1†^, Mincheol Jang^2†^, Ji-Ho Park^2,3*^, and Hyun Jung Chung^1*^

^1^ Department of Biological Sciences, Korea Advanced Institute of Science and Technology (KAIST), 291, Daehak-ro, Yuseong-gu, Daejeon, 34141, Republic of Korea

^2^ Department of Bio and Brain Engineering, Korea Advanced Institute of Science and Technology (KAIST), 291, Daehak-ro, Yuseong-gu, Daejeon, 34141, Republic of Korea

^3^ KAIST Institute for Health Science and Technology, Korea Advanced Institute of Science and Technology (KAIST), 291, Daehak-ro, Yuseong-gu, Daejeon, 34141, Republic of Korea

E-mail: hyunjc@kaist.ac.kr; jihopark@kaist.ac.kr

^†^ These authors contributed equally:

^*^ Corresponding authors

**Table S1.** Primers for targeted deep sequencing to determine gene editing efficiencies, according to different sgRNAs targeting IL-10.

^
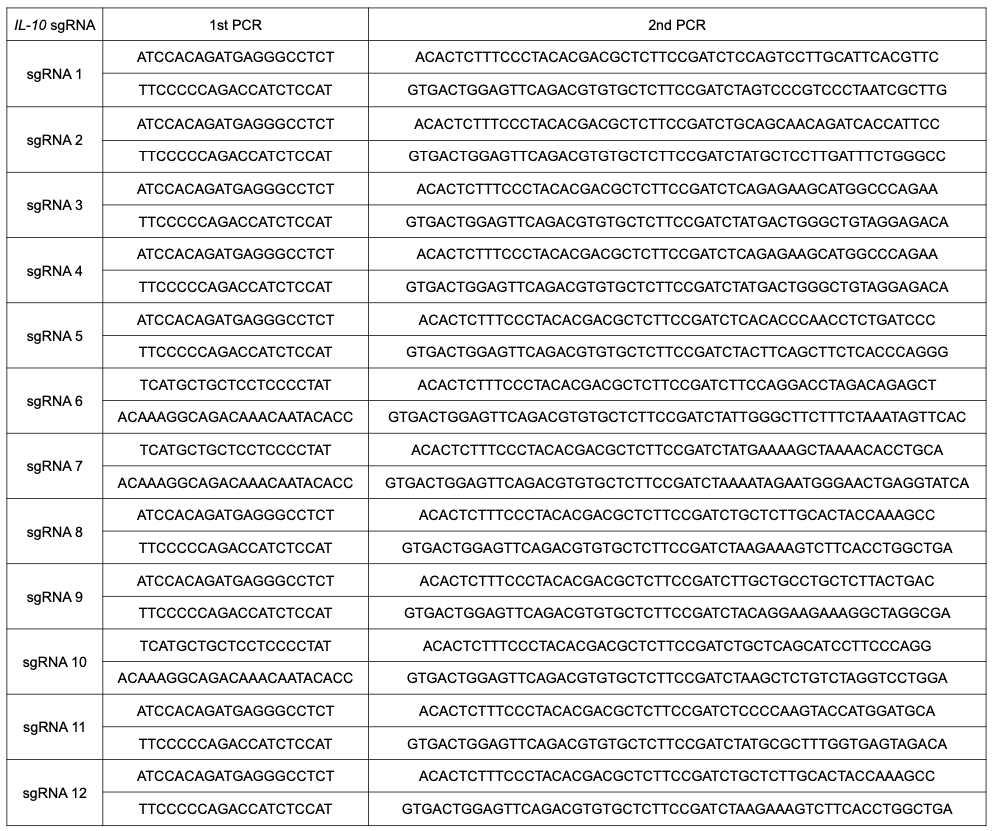
^

**Table S2.** Primers for targeted deep sequencing to determine off-target effects, according to different sgRNAs targeting IL-10.

**
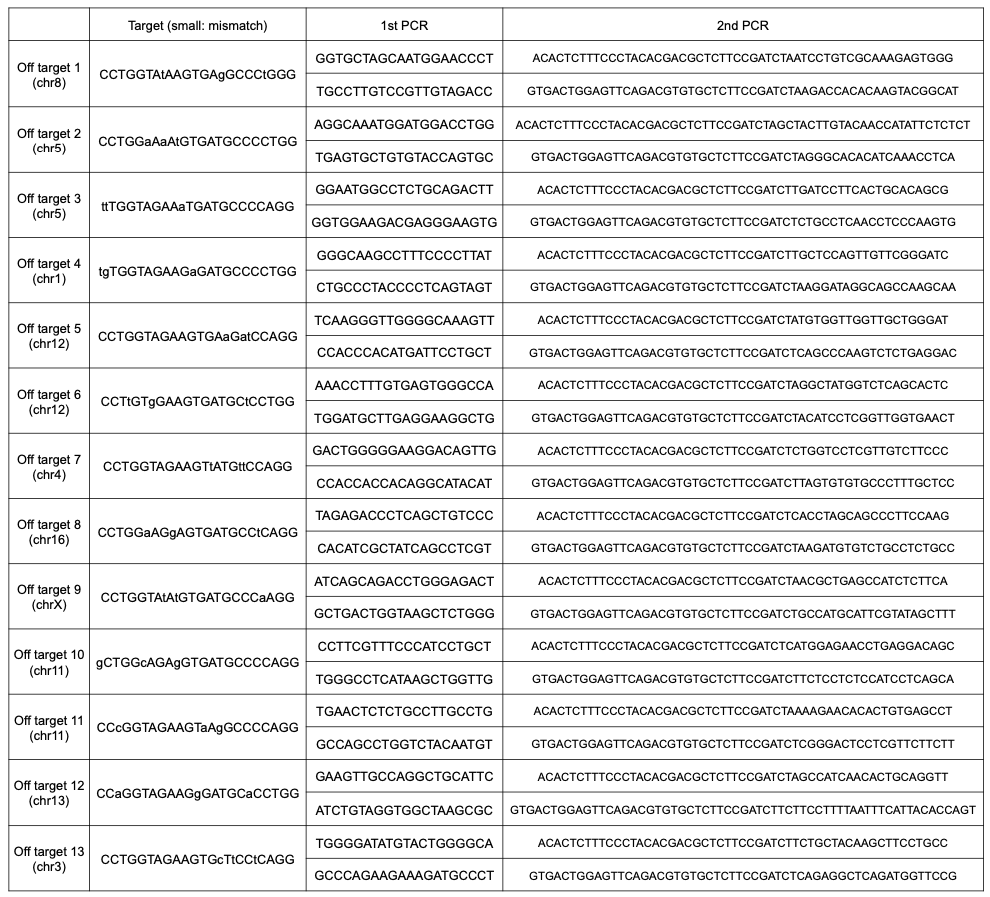
**

**
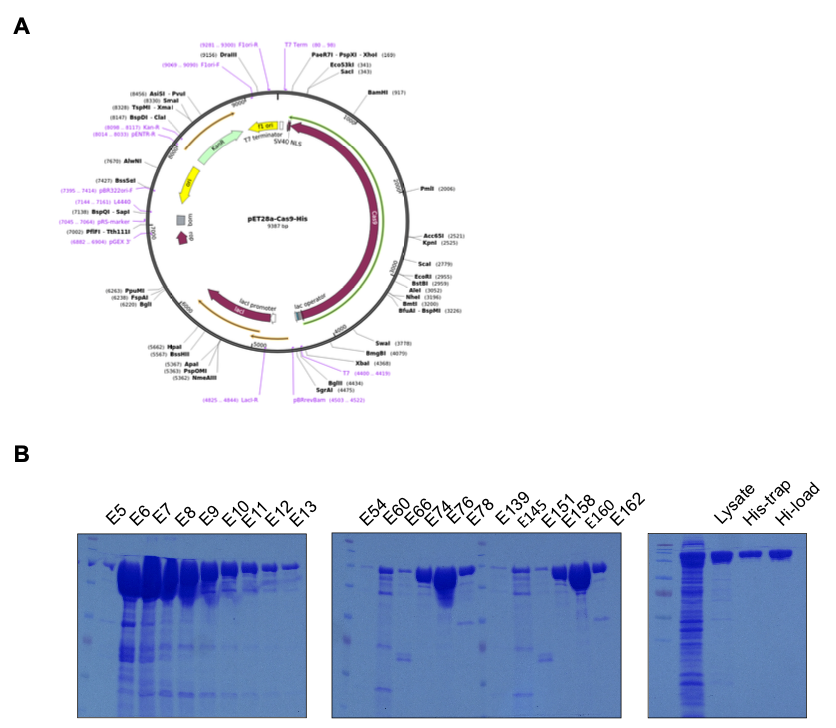
**

**Figure S1.** Purification of Cas9. **A** Plasmid map of Cas9 expression plasmid. **B** SDS-PAGE of eluents at each step during Cas9 purification.

**
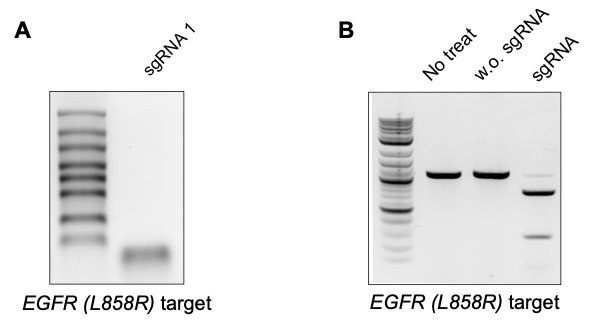
**

**Figure S2.** Synthesis and characterization of sgRNA targeting EGFR (L858R) of the surrogate reporter. **A** sgRNA was synthesized by IVT. **B** DNA cleavage activity of sgRNA by treating RNP to target DNA for 90 min, and analysis of cleaved products by gel electrophoresis.

**
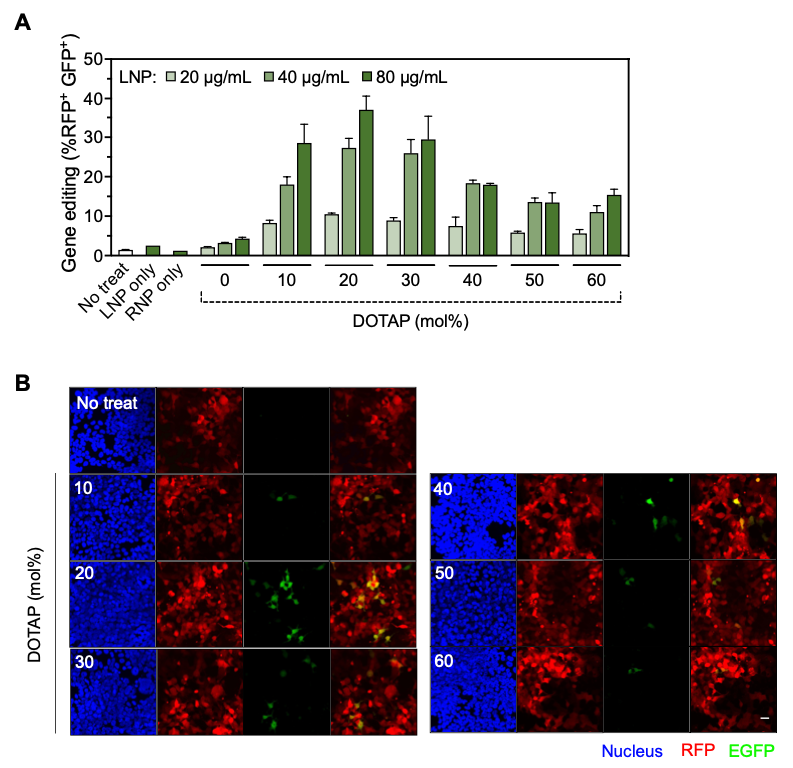
**

**Figure S3.** Gene editing by LNPs added with DOTAP in HEK293T surrogate reporter cell line. **A** Flow cytometry and **B** confocal microscopy of treated cells according to various molar ratios of DOTAP. Scale bar, 40 µm.

**
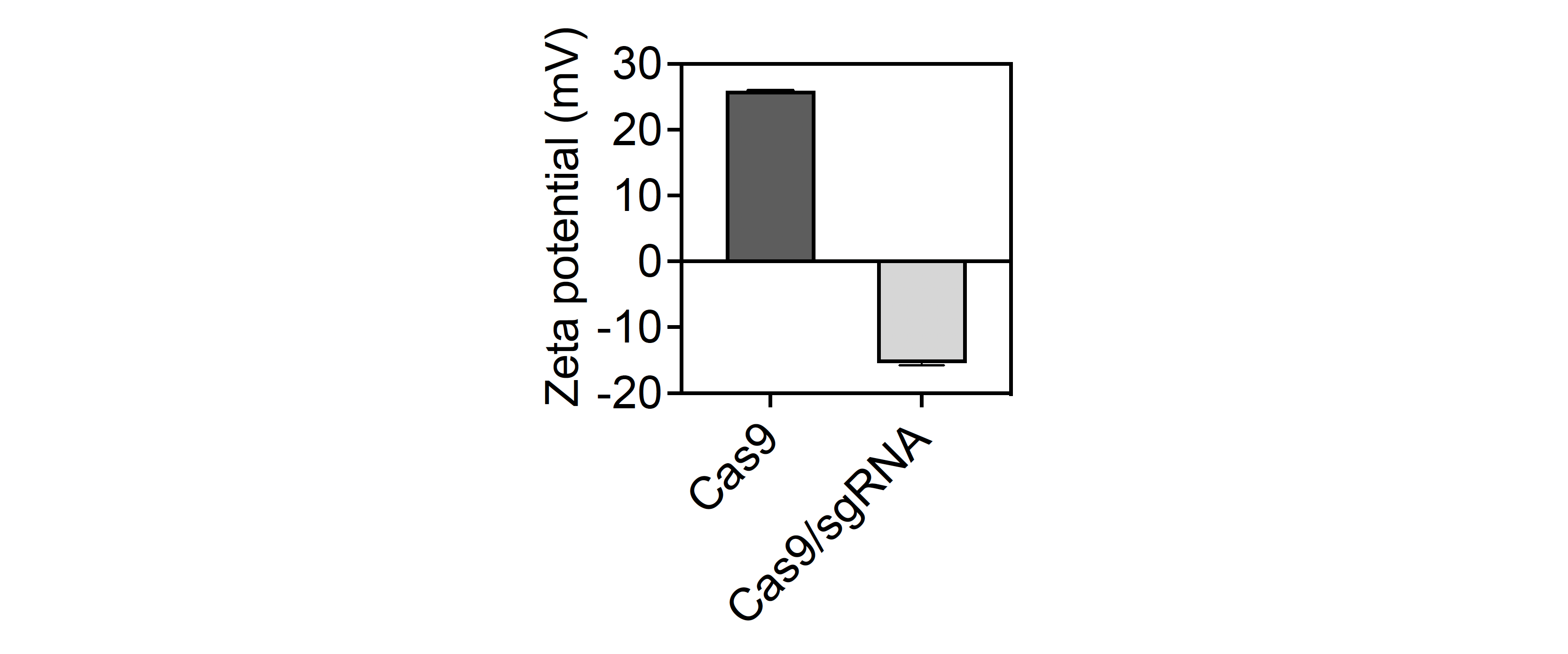
**

**Figure S4.** Zeta potential of Cas9 and Cas9/sgRNA (n = 3).

**
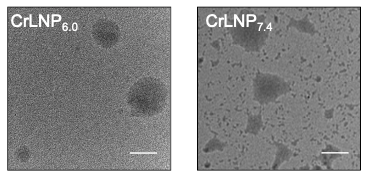
**

**Figure S5.** TEM of CrLNPs prepared at different pHs (pH 6.0 and 7.4). Scale bar, 100 nm.

**
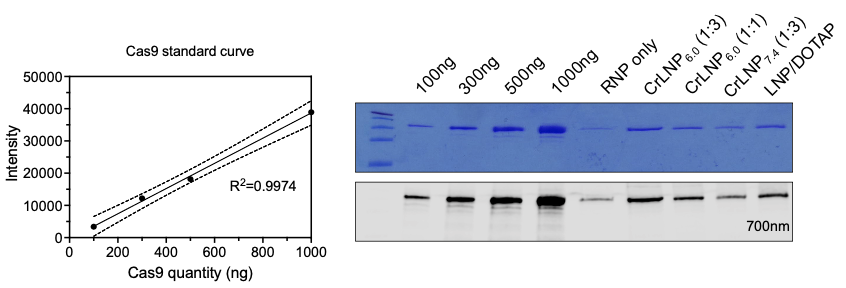
**

**Figure S6.** Loading efficiency of Cas9 in CrLNPs. Standard curve and SDS-PAGE of AF647-conjugated Cas9 after loading.

**
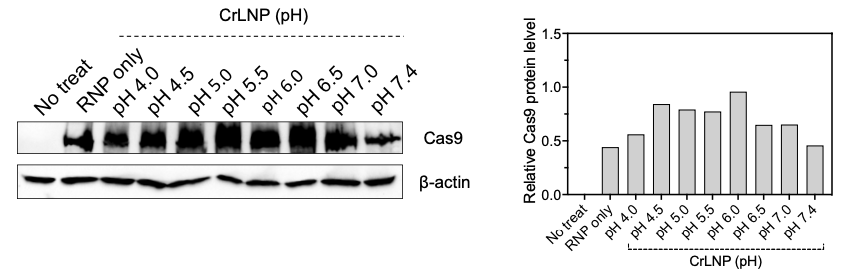
**

**Figure S7.** Western blot of Cas9 after treating HEK293T cells with CrLNP prepared at different pHs. Cells were treated for 6 h, and the total proteins were blotted on PVDF membranes and analyzed by measuring relative Cas9 protein levels normalized to actin levels.

**
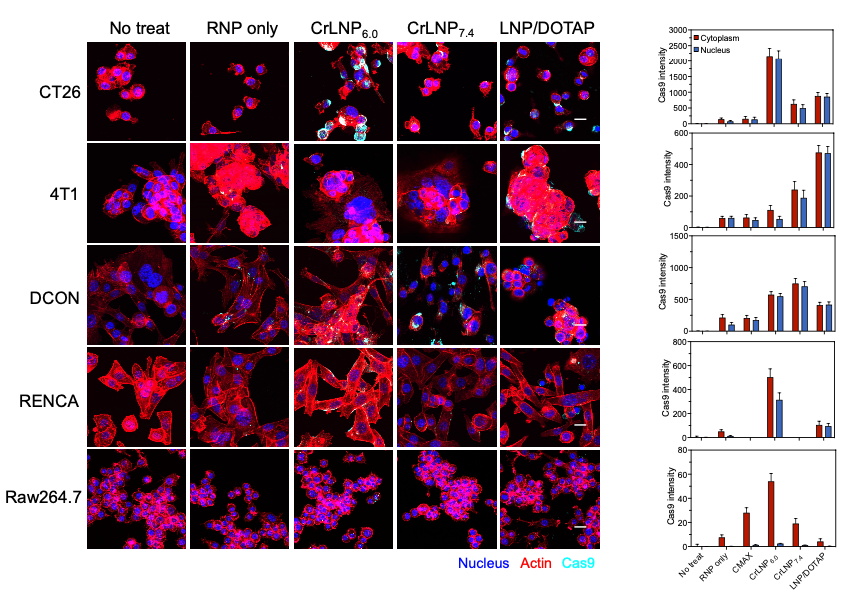
**

**Figure S8.** Confocal images and quantification of various cells (CT26, 4T1, DCON, RENCA, Raw264.7) treated with CrLNP for 6 h. Scale bar 40 µm.

**
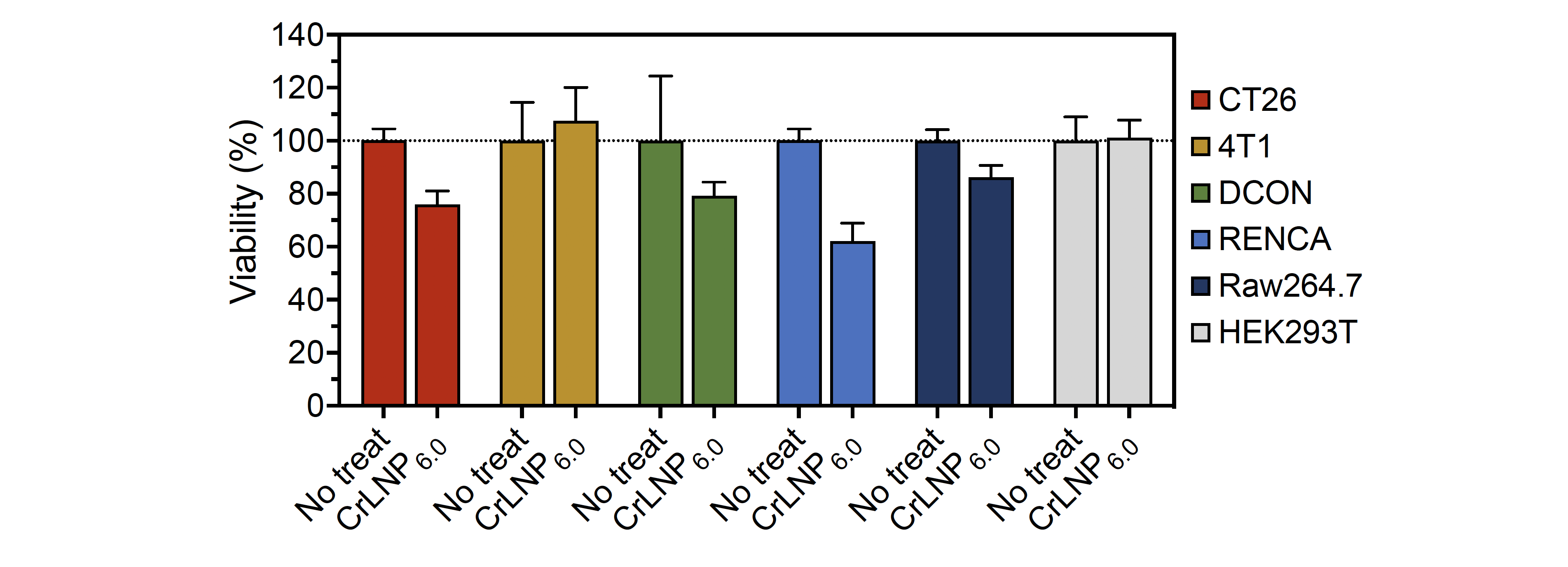
**

**Figure S9.** Cytotoxicity of CrLNP_6.0_ treated at 20 µg/mL for 6h in various cell lines (n = 3).

**
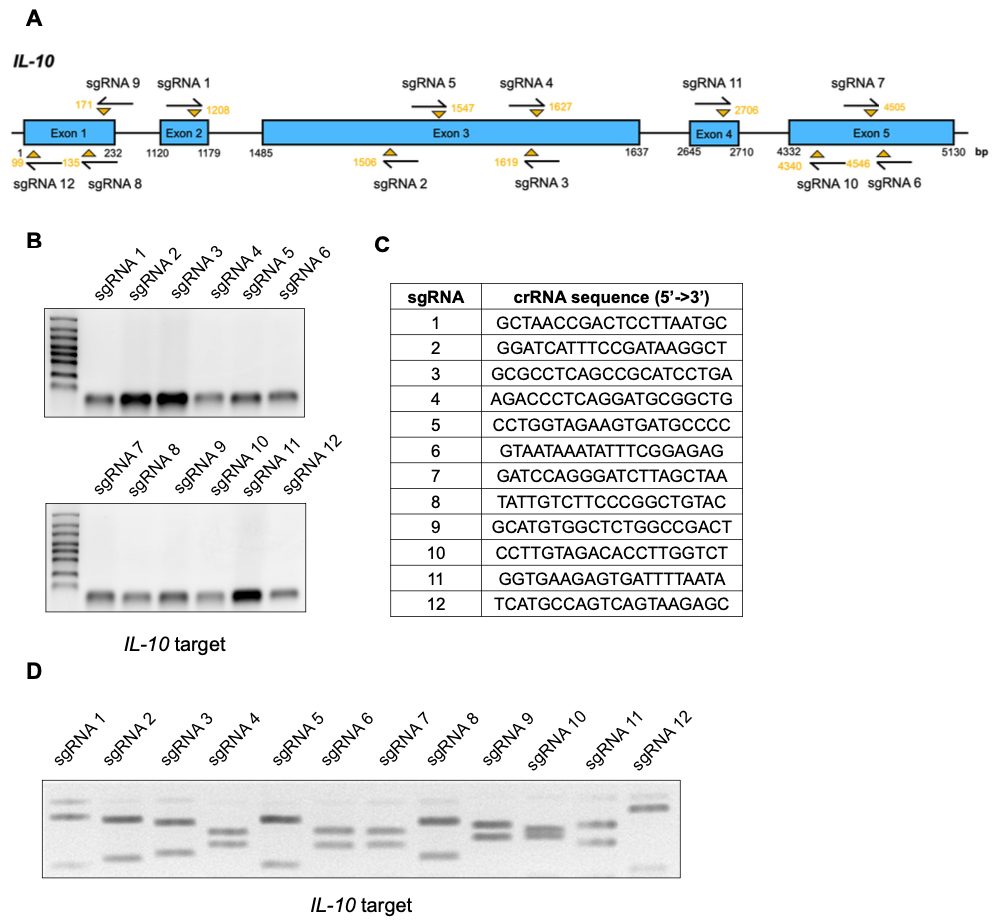
**

**Figure S10.** Synthesis and characterization of sgRNA targeting IL-10. **A** Scheme of IL-10 gene aligned with the designed sgRNAs targeting 12 different regions. **B** Synthesis of sgRNAs by IVT. **C** Table showing the list of 12 sgRNAs targeting IL-10. **D** Cleavage activity of sgRNAs by treating RNPs to IL-10 target DNA for 90 min, and analyses of cleaved products.

**
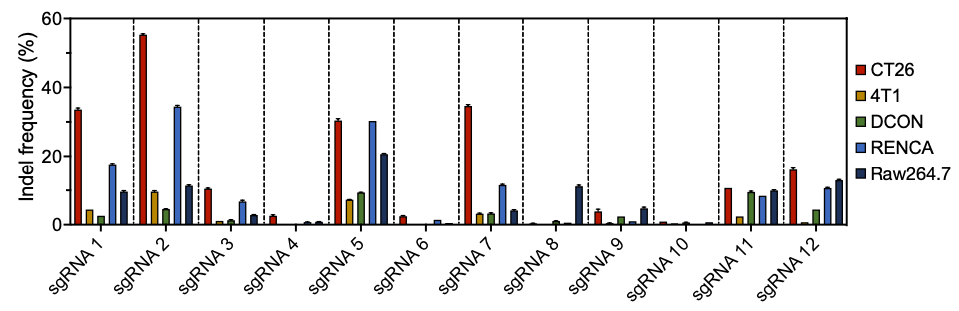
**

**Figure S11.** Gene editing efficiencies by treating different sgRNAs targeting IL-10 to various cell lines for 72 h, and analyses by targeted deep sequencing.

**
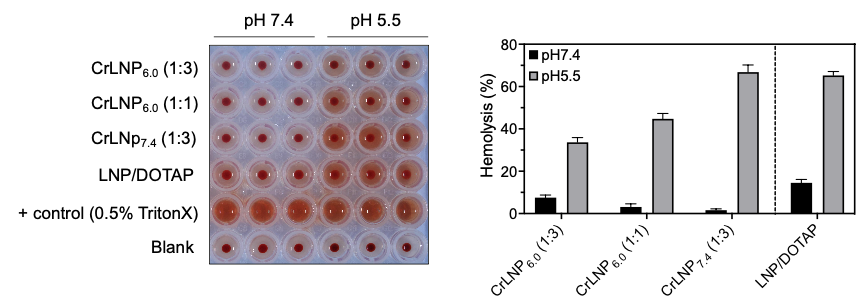
**

**Figure S12.** Hemolysis assay by treating CrLNPs to human red blood cells for 1 h, and measuring the changes in absorbance.

**
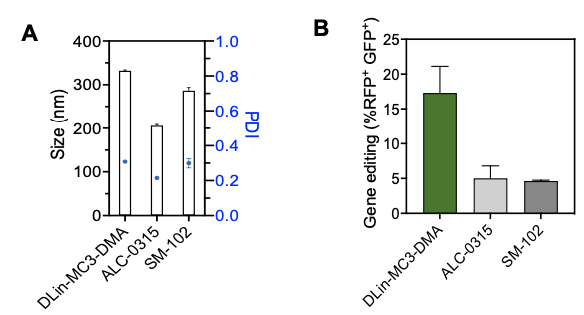
**

**Figure S13.** Evaluation of CrLNP_6.0_ prepared with various ionizable lipids. **A** Hydrodynamic size and **B** gene editing efficiency by treating to HEK293T surrogate reporter cells.

*
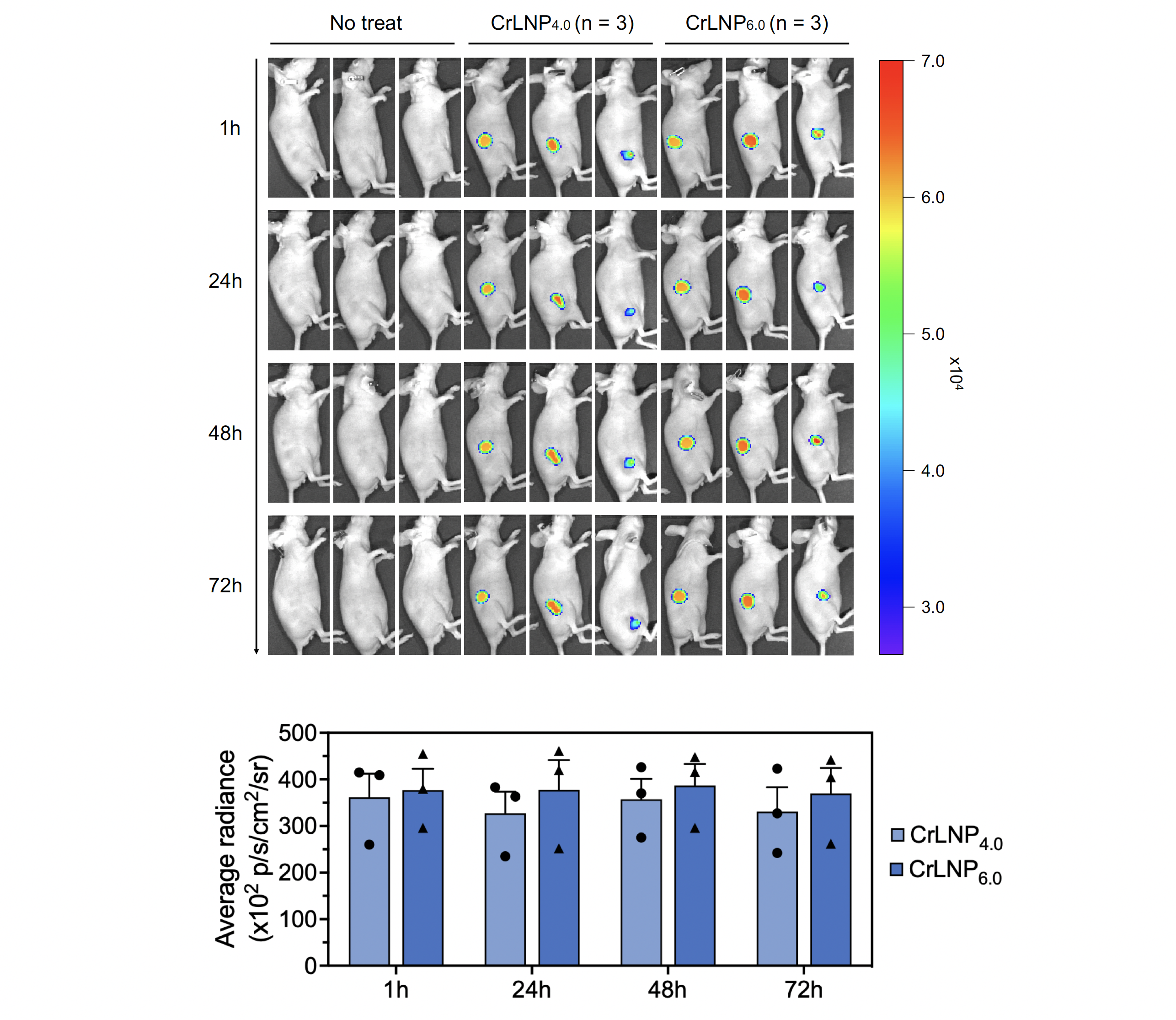
*

**Figure S14.** IVIS imaging and quantification of AF647-conjugated Cas9 signals at different time points after intratumoral injection of CrLNP_4.0_ and CrLNP_6.0_ (n = 3). Data are shown as mean ± SD.

**
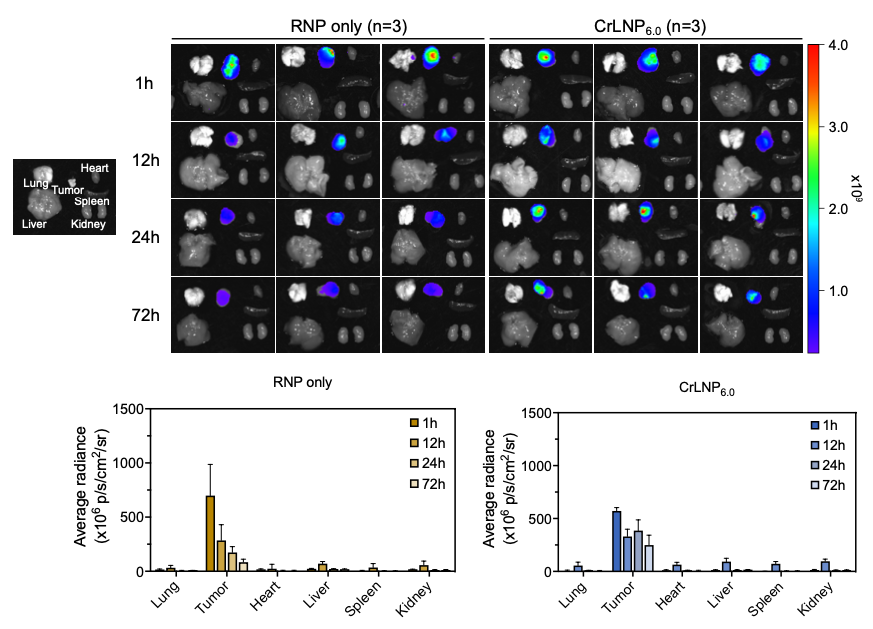
**

**Figure S15.** Biodistribution upon intratumoral injection of CrLNPs. Mice were sacrificed after each time point and signals of AF647-conjugated Cas9 were imaged using IVIS (n = 3).

**
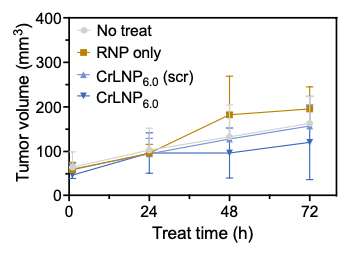
**

**Figure S16.** Measurement of tumor volumes after injection of CrLNP_6.0_ (n = 4).

**
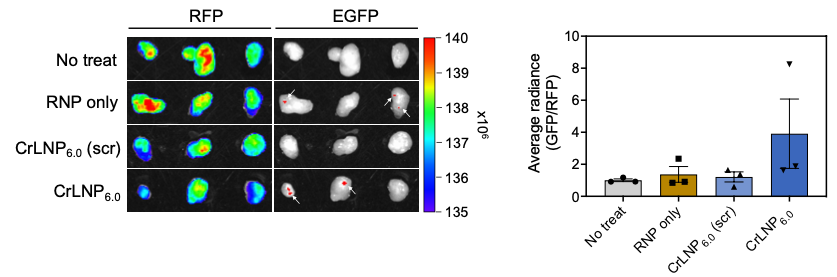
**

**Figure S17.** IVIS imaging of HEK293T tumors after treatment with CrLNP_6.0_. Signals of the surrogate reporter (EGFP) were analyzed by excising tumors 72 h after intratumoral injection (n = 3).


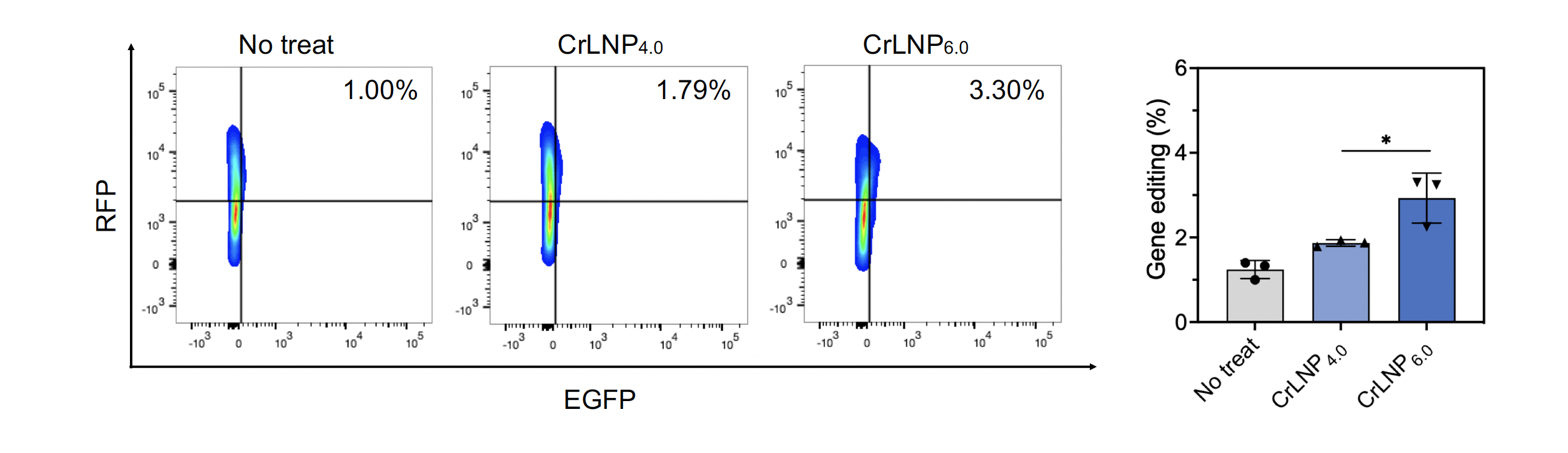


**Figure S18.** In vivo gene editing by treatment of CrLNP_4.0_ and CrLNP_6.0_. Scatter plot of a representative sample from each group, and mean values for each group (n = 3). Data are shown as mean ± SD, *P < 0.05 by one-way ANOVA.
